# A Systematic Review on *HOX* Genes as Potential Biomarkers in Colorectal Cancer: An Emerging Role of *HOXB9*

**DOI:** 10.3390/ijms222413429

**Published:** 2021-12-14

**Authors:** Eirini Martinou, Giulia Falgari, Izhar Bagwan, Angeliki M. Angelidi

**Affiliations:** 1 Hepatobiliary and Pancreatic Surgery Department, Royal Surrey County Hospital, Guildford GU2 7XX, UK; 2 Faculty of Health and Medical Sciences, University of Surrey, Guildford GU2 7XH, UK; falgari.g@gmail.com; 3 Department of Histopathology and Molecular Biology, Royal Surrey County Hospital, Guildford GU2 7XX, UK; izhar.bagwan@nhs.net; 4 Department of Medicine, Beth Israel Deaconess Medical Centre, Harvard Medical School, Boston, MA 02215, USA

**Keywords:** homeobox, HOX, colorectal, cancer, adenocarcinoma

## Abstract

Emerging evidence shows that Homeobox (*HOX*) genes are important in carcinogenesis, and their dysregulation has been linked with metastatic potential and poor prognosis. This review (PROSPERO-CRD42020190953) aims to systematically investigate the role of *HOX* genes as biomarkers in CRC and the impact of their modulation on tumour growth and progression. The MEDLINE, EMBASE, Web of Science and Cochrane databases were searched for eligible studies exploring two research questions: (a) the clinicopathological and prognostic significance of HOX dysregulation in patients with CRC and (b) the functional role of *HOX* genes in CRC progression. Twenty-five studies enrolling 3003 CRC patients, showed that aberrant expression of HOX proteins was significantly related to tumour depth, nodal invasion, distant metastases, advanced stage and poor prognosis. A post-hoc meta-analysis on HOXB9 showed that its overexpression was significantly associated with the presence of distant metastases (pooled OR 4.14, 95% CI 1.64–10.43, I^2^ = 0%, *p* = 0.003). Twenty-two preclinical studies showed that HOX proteins are crucially related to tumour growth and metastatic potential by affecting cell proliferation and altering the expression of epithelial-mesenchymal transition modulators. In conclusion, HOX proteins may play vital roles in CRC progression and are associated with overall survival. HOXB9 may be a critical transcription factor in CRC.

## 1. Introduction

Colorectal Cancer (CRC) is the most common gastrointestinal malignancy and the third leading cause of cancer-related death worldwide [1,2]. Despite significant advances in diagnostic and therapeutic strategies, the prognosis for CRC patients remains poor, indicating that cancerous cells are not entirely eradicated by current therapies, thus leading to metastatic disease which is the primary cause of cancer-related mortality [3]. CRC arises as a result of the accumulation of genetic and epigenetic changes, which transform normal glandular epithelial cells into invasive carcinomas and eventually progress into metastatic disease [4]. Colorectal carcinogenesis is a complex multistep process involving the dysregulation of oncogenes or tumour suppressor genes related to initiation, progression and resistance to therapy [4,5]. Therefore, there is an urgent need to identify novel biomarkers which could be used to predict prognosis and act as therapeutic targets.

**Figure 1:**
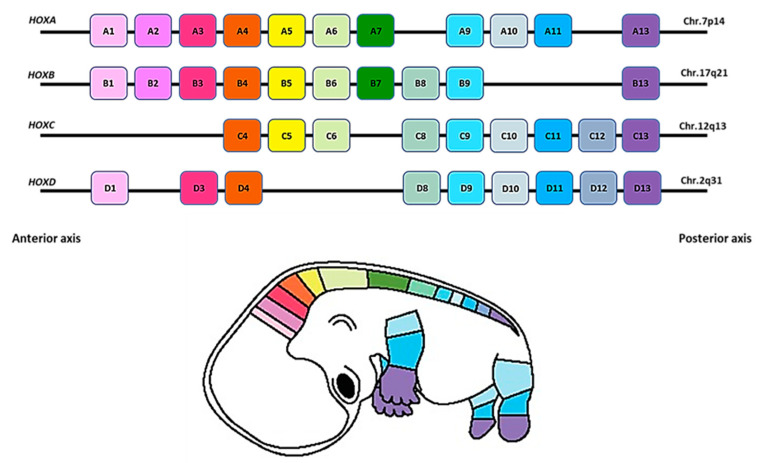
*HOX* genes in the human genome. Adapted from Durston et al. [8] The colour coding represents the correspondence between the genomic order of each *HOX* gene in the chromosomal cluster and the segmental identity in a human embryo (Microsoft PowerPoint software was used to create this figure).

In recent decades, a lot of attention has been paid to the role of homeobox (*HOX*) genes in cancer [6]. *HOX* genes encode a highly conserved family of homeodomain-containing transcription factors which play essential roles in embryonic development including morphogenesis, organogenesis and differentiation [7]. HOX proteins control various cellular processes by regulating the expression of several downstream target genes; hence they can alter cell behaviour such as proliferation, invasion and migration [7]. The human genome contains 39 *HOX* genes which are classified into four clusters, *HOXA*, *HOXB*, *HOXC* and *HOXD* based on their sequence similarity and chromosomal position, as shown in Figure 1 [8]. *HOX* genes are characterized as master regulators during development and alterations in their expression leads to developmental abnormalities and has been reported to be associated with an increased incidence of malignant tumours in humans [9,10]. Numerous studies have shown that *HOX* genes can act either as oncogenes or tumour suppressors depending on cancer type. For instance, *HOXA9* has an oncogenic role in acute leukaemia whereas it acts as a tumour suppressor in breast cancer by regulating the expression of Breast Cancer gene 1 (*BRCA1*) [11,12]. Additionally, *HOXB9* has been shown to inhibit cancer cell proliferation in gastric carcinoma whilst it demonstrates an oncogenic role in breast cancer [13,14].

Emerging evidence shows that HOX transcription factors have significant contributions in the hallmarks of cancer and especially in the proliferative advantage as well as in the invasion and metastasis element, and therefore may play important roles in tumour progression [15]. Expression of *HOX* genes is dysregulated and often reported to be associated with aggressive nature of tumour biology and poor survival in various types of cancers [16,17,18,19]. In CRC, several studies have revealed that some *HOX* genes demonstrate aberrant expression in cancerous tissues suggesting that they be considered as potential biomarkers. Although, there is increasing evidence that *HOX* genes may be important in CRC, a systematic understanding regarding their role in CRC progression and their clinicopathological significance is still lacking. To better understand the existing evidence with regard to the prognostic and functional role of *HOX* genes in CRC, the authors performed a systematic review of the current literature. Specifically, this review aims to answer the following research questions: (a) What are the clinicopathological and prognostic significance of *HOX* genes dysregulation in CRC and (b) What is the functional role of *HOX* genes in CRC progression?

## 2. Materials and Methods

### 2.1. Search Strategy

A literature search was conducted for eligible studies in the Medline (https://pubmed.ncbi.nlm.nih.gov/advanced/, 12 July 2020), EMBASE (https://www.embase.com, 19 July 2020), Web of Science (https://www.webofknowledge.com, 25 July 2020) and Cochrane Database (https://www.cochranelibrary.com/, 2 August 2020) search engines in accordance with the Preferred Reporting Items for Systematic Reviews and Meta-Analyses (PRISMA) guidelines [20]. The end date of the retrieval period was the 1 July 2021. A search strategy was developed in Medline (https://pubmed.ncbi.nlm.nih.gov/advanced/, 31 March 2020) including keywords, Medical Subject Headings (MESH) and synonyms related to *HOX* genes, colorectal and neoplasms. The strategy was adapted for the other databases using separate algorithms for each search engine (supplementary material, ESM_1). This systematic review was registered in the international prospective register of systematic reviews (PROSPERO, https://www.crd.york.ac.uk/prospero/) with the identification number CRD42020190953 (registration date was 29 June 2020).

### 2.2. Eligibility Criteria and Study Selection

Three reviewers (EM, GF and AA) independently selected and identified eligible English language studies based on predefined inclusion criteria according to the research question. Discrepancies between the three reviewers were resolved by discussion or with a 4th author.

For the first research question, studies conducted on individuals over 18 years old with sporadic colorectal adenocarcinoma and reporting on *HOX* genes were included. Specifically, studies reporting at least one of the following criteria were included: (i) studies on *HOX* dysregulation between cancer and normal tissue, (ii) studies on the association of *HOX* genes with clinicopathological characteristics of CRC, and (iii) studies reporting outcome measures such as OS and DFS. Exclusion criteria were studies reporting the dysregulation of *HOX* genes in cell lines or animal tissues. Comparison groups were selected to be the following: cancer to normal colorectal tissue as well as high versus low *HOX* gene expression patient group. Outcomes were defined as: *HOX* gene dysregulation, tumour depth, lymph node status, metastases, stage of colorectal cancer disease, grade of disease, disease free survival (DFS) and overall survival (OS) rates.

For the second research question, laboratory-based and animal research studies were included reporting on the effect of *HOX* gene expression in CRC cell growth. The intervention was considered gene expression editing to either suppress or overexpress the *HOX* gene of interest. The intervention group in the included studies consisted of either human colorectal cancer cell lines or animal models which had an altered expression of *HOX* genes. Therefore, the comparison groups were defined as edited versus non-edited human CRC cell lines or animal models. Outcomes were selected to be cell proliferation, cell migration, cell invasion in vitro as well as tumour growth and metastases in vivo.

Studies such as case reports, editorials, opinions, conference abstracts, reviews and other secondary research studies were excluded. Studies not using the universal *HOX* chromosomal cluster terminology as described in Figure 1 for reporting findings were also excluded for both research questions.

### 2.3. Data Extraction, Synthesis and Quality Assessment

For each study, the following details were extracted on publication year, the surname of the first author, study design, participant characteristics (sample size, gender), study characteristics (intervention and control group, endpoint assays) and outcomes. The quality of each eligible primary study involving human participants was assessed using the National Heart, Lung and Blood Institute (NIH) study quality assessment tools for case studies, available online at https://www.nhlbi.nih.gov/health-topics/study-quality-assessment-tools (last accessed 20 December 2020), (Table S1). Preclinical animal studies were assessed using the Systematic Review Centre for Laboratory animal experimentation risk of bias tool (SYRCLE’S), (Table S2) [21,22]. For cell line studies, no established quality assessment tool is currently available [21]. The results were summarised narratively according to each research question using a qualitative data synthesis approach. We conducted a post-hoc random-effects meta-analysis to summarise effect estimates when at least two peer-reviewed studies were available and were sufficiently homogenous in terms of subjects involved, interventions and outcomes [23]. Results were expressed as odds ratio (OR) with 95% confidence intervals (CIs) using Review Manager Version 5.4.1 (Cochrane collaboration) I^2^ statistics and *p*-value were calculated to assess the heterogeneity between the studies. Forest plots were produced to present the ORs with 95% CIs, the percentage weight and the heterogeneity between studies included in each meta-analysis.

## 3. Results

### 3.1. Study Selection

**Figure 2:**
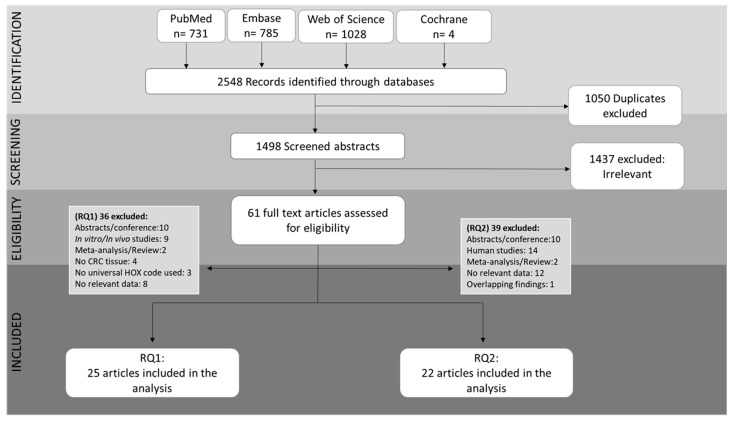
PRISMA flow chart of systematic review article retrieval (Microsoft PowerPoint software was used to create this figure).

The initial literature search identified 2548 eligible citations. Following duplicate citation removal 1498 studies were screened for eligibility. For research questions 1 and 2, a total of 25 and 22 studies met our inclusion criteria, respectively and were included in the final analysis. The process of literature retrieval, according to PRISMA guidelines, is shown in detail in Figure 2. Study characteristics and findings are presented for each research question separately.

### 3.2. Clinicopathological Characteristics and Prognostic Significance of HOX Dysregulation in CRC

#### 3.2.1. Study Characteristics

A total of 3003 patients with stage I–IV CRC between 20 and 90 years old were included in the studies [24,25,26,27,28,29,30,31,32,33,34,35,36,37,38,39,40,41,42,43,44,45,46,47,48]. All 25 studies were single-centre and were published between 1997 and 2020. Two studies included patients who either received or did not receive neoadjuvant chemotherapy [42,43], five studies included only patients without neoadjuvant chemotherapy [36,38,44,45,47] whereas in the remaining studies no information on the chemotherapy status was available. Differential *HOX* gene expression between cancer and normal tissue was reported by 22 studies [24,25,26,27,28,29,31,32,33,34,35,36,37,38,39,40,41,43,44,46,48]. The analysis assays were real-time quantitative polymerase chain reaction [RT-qPCR], immunohistochemistry [IHC] and Western blotting [WB]. Four studies included bioinformatics analysis from publicly available RNA sequencing data [25,28,39,41], as shown in Table 1.

Seventeen studies investigated the association of *HOX* genes with clinicopathological characteristics in patients with CRC [24,26,27,28,30,35,36,37,38,40,42,43,44,45,46,47,48], Table 1. Variables included at least one of the following: Age, sex, tumour depth, lymph node status, metastases, stage of colorectal cancer disease, grade of disease and carcinoembryonic antigen (CEA) levels.

The impact of HOX protein expression on OS and DFS in patients with CRC was investigated by 12 [24,25,26,28,36,38,39,40,43,46,47,48] and 4 [28,35,36,42] studies, respectively, as shown in Table 1. OS and DFS rates in patients with high versus low expression of the HOX protein of interest were compared. Most of the studies used a semiquantitative IHC approach to score the expression levels of HOX proteins, whereas two studies used the median mRNA level as a cut-off value [25,28].

#### 3.2.2. Findings

**Figure 3:**
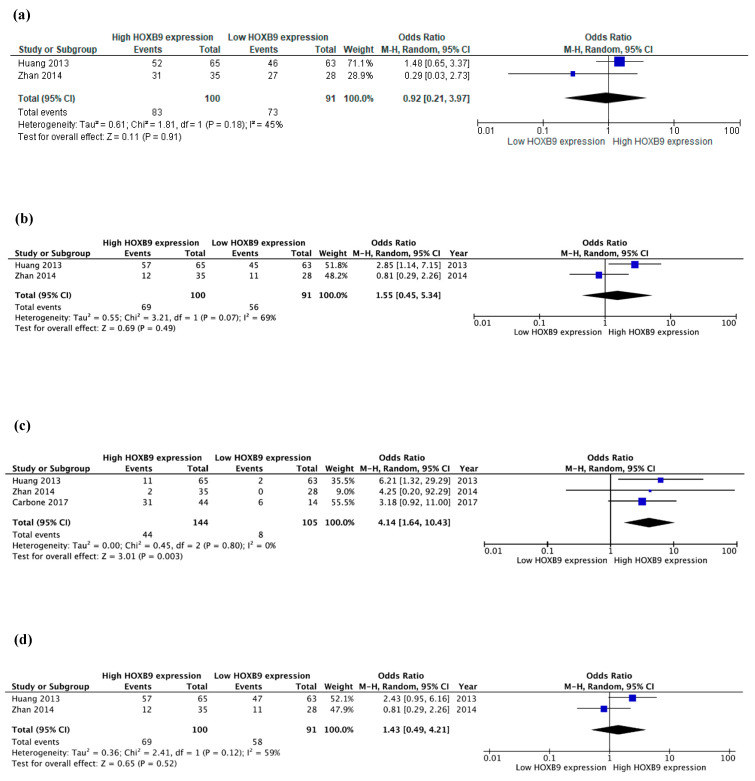
Forest plots of included trials assessing the effect of HOXB9 expression on (**a**) tumour depth, (**b**) lymph node invasion, (**c**) presence of metastatic disease, (**d**) advanced stage of CRC. Odds ratios and 95% confidence intervals (95% CIs), were pooled using random-effect meta-analysis. Blue squares indicate the effect size for each study (ORs between high and the low HOXB9 expression group) and the length of the lines indicate the 95% CIs. The size of the square represents its weight in the analysis. The black diamond on the bottom of the forest plot indicates the overall weighted effect size. I^2^ indicates between-study heterogeneity. Detailed characteristics of each study are available in Table 1 of the main manuscript (M-H: Mantel Haenszel, CI: confidence intervals).

Regarding *HOX* gene dysregulation and its clinicopathological significance in CRC, 26 out of 39 *HOX* genes were identified in this systematic review to be differentially expressed in cancerous versus normal colon tissues with 15 of them being overexpressed and six being downregulated, as shown in Table 1 [28,31,32,37,38,39,44,45,46,47,48]. Discrepancies were reported between studies for five *HOX* genes (*A4*, *B8*, *B9*, *B13* and *D10*). Among the dysregulated *HOX* genes and their protein products, several were found to demonstrate potential clinical significance in CRC. Three studies reported that patients with high HOX (B7, B9, C6) protein expression levels demonstrated significantly advanced T status in comparison with patients with low expression [26,42,43] (Table 1). Post-hoc meta-analysis for HOXB9 protein found no statistical association between high HOXB9 and tumour depth (OR 0.92, 95% CI: 0.21–3.97, *p* = 0.910), (Figure 3a). Similarly, regarding N status, six studies showed that the percentage of patients with regional lymph node invasion was significantly increased in the high HOX protein expression group for the following: HOXA3, A9, B8, B9, B13, C6, D1 and D9 [24,27,37,40,43,48]. Post-hoc meta-analysis for HOXB9 revealed no statistical association between high HOXB9 and lymph node invasion (OR 1.55, 95% CI: 0.45–5.34, *p* = 0.490), (Figure 3b). Inverse correlation between HOX expression and nodal invasion was demonstrated only for HOΧD10 by Wang et al. [44]. Eight studies investigated the presence of metastatic disease according to HOX expression levels [28,40,42,43,45,46,47,48]. Two studies by Liao et al. [26] and Huang et al. [48] for HOXB7 and B9, respectively, reported that significantly more patients had metastatic disease in the high HOX expression group in comparison with the low expression one (Table 1). Three studies (Huang et al., Zhan et al. and Carbone et al.) were included in the post-hoc meta-analyses regarding HOXB9 and the presence of metastatic disease. High HOXB9 expression was found to be associated with a significant risk for metastatic disease (OR 4.14, 95% CI: 1.64–10.43, *p* = 0.003), (Figure 3c). Findings remained significant when sensitivity analyses were performed by excluding one study each time (data not shown herein). Eleven studies have reported findings regarding the stage of the disease and level of HOX expression [24,26,28,30,35,36,38,40,45,47,48]. For HOXA3, A9, B7 and D9, high expression groups correlated with an increased number of patients with advanced disease in comparison with the low expression groups [24,26,36,40]. For HOXB9 studies by Zhan et al. [47] and Huang et al. [48] reported antithetical findings (Table 1). For HOXB9, post-hoc meta-analysis showed no statistical correlation between advanced stage and high HOXB9 expression (OR 1.43, 95% CI: 0.49–4.21, *p* = 0.520), (Figure 3d). Most studies reported no significant association between tumour differentiation and HOX expression level, except for D9, where Liu et al. reported that high HOXD9 levels were significantly associated with poor differentiation [24]. The studies by Hoshino et al. [46] and Zhan et al. [47] reported contradictory results regarding HOXB9 expression levels and their association with CRC differentiation (Table 1). Given the limited available data, it appears that there is no significant association between age, sex and CEA with HOX expression levels.

The prognostic role of HOX dysregulation (A3, B7, B8, C6, C11, D9, D10) was investigated by 14 studies [24,25,26,28,35,36,38,39,40,42,43,46,47,48]. Specifically, patients who were characterised by high HOX expression levels had significantly worse survival rates in comparison with the ones with low expression levels. Only two studies by Ji et al. [43] and Liao et al. [26] conducted multivariable analysis and reported that HOXC6 and HOXB7 are independent prognostic markers in patients with CRC (Table 1). HOXB9 was investigated by four studies that reported contradictory results with regard to its positive or negative impact on survival [38,46,47,48]. In terms of DFS, studies showed that high expression levels of HOXA3, A10, B8 and B9 were significantly associated with worse DFS rates in patients with CRC [28,35,36,42]. HOXA10 and B9 were additionally reported to serve as independent risk factors for worse DFS by Yuan et al. [35] and Carbone et al. [42], respectively, in a multivariable analysis model (Table 1).

### 3.3. Functional Role of HOX Genes in CRC Progression

#### 3.3.1. Study Characteristics

Twenty-two studies investigated the functional role of *HOX* genes dysregulation in CRC progression [24,25,26,28,31,34,35,36,40,41,43,46,47,48,49,50,51,52,53,54,55,56] as shown in Table 2 and Table 3, Tables S3 and S4. All studies were preclinical with 11 having conducted in vivo as well as in vitro experiments. All in vitro experimental studies used various human colorectal cell lines to conduct gain and/or loss of function experiments by altering the gene expression level of the *HOX* gene of interest. In vivo studies used nude mice which were subjected to subcutaneous injection of human CRC cell lines with altered or not *HOX* expression levels [24,26,28,35,36,43,46,47,48,55,56].

Twenty studies investigated the impact of *HOX* genes dysregulation on tumour growth, eight of which performed additional in vivo experiments [24,25,26,28,31,34,35,36,40,41,43,46,47,49,50,51,52,53,54,56]. The in vitro primary outcome was the cell proliferation rate over time being measured by relevant assays, as shown in Table 2 and Table 3, Tables S3 and S4. The in vivo primary outcome was tumour growth which was assessed differently in each study by reporting either tumour weight (gr), size (diameter in cm) or volume (mm^3^), (Table 2).

Ten studies investigated the effect of *HOX* genes differential expression in the metastatic potential in CRC [24,28,34,41,47,48,49,50,52,55]. The primary outcomes were the percentage of cells that showed invasion and/or migration in the relevant assays between the control versus the intervention group. Secondary outcomes were molecular markers involved in the Epithelial-Mesenchymal Transition (EMT), being reported by six studies [24,34,41,48,52,55]. Five studies provided additional results from in vivo experiments by assessing the number of lung/liver metastases as the primary outcome [24,28,47,48,55], (Table 2). One study reported markers involved in angiogenesis and vessel formation in vivo [46].

#### 3.3.2. Findings

Regarding *HOX* dysregulation and tumour growth in CRC, of the eighteen *HOX* genes and their protein products that have been investigated to date, fifteen were found to have oncogenic properties whereas only three were reported to exert tumour suppressive functions. Specifically, loss of function in vitro studies showed that knockdown of *HOXA1*, *A3*, *A4*, *A9*, *A10*, *B8*, *C6*, *C11*, *C13* and *D3* resulted in a reduced proliferation rate of CRC cells [25,28,35,36,40,43,50,51,53,54]. Additionally, overexpression in vitro experiments showed that increased levels of *HOXA6*, *B2*, *B7* and *D9* resulted in increased proliferation rates indicating the tumour promoting properties of the above gene products [24,26,34,49]. The findings from in vivo studies, which have been conducted for *HOXA3*, *B7*, *B8*, *B13*, *C6* and *D9*, agreed with the functional role observed in vitro [24,26,28,36,43,56]. Findings for *HOXB9* by Hoshino et al. [46] and Zhan et al. [47] report contradictory results, with the former study reporting tumour promoting and the latter showing tumour-suppressive properties in CRC.

Regarding *HOX* dysregulation and metastatic potential in CRC, nine *HOX* genes and their proteins have been reported to affect CRC disease progression in vitro [24,28,34,41,47,48,49,50,52,55]. The knockdown of *HOXA1* and *HOXB8* resulted in a decreased number of invasive cells [28,50]. Similarly, the overexpression of *HOXA6*, *B2* and *D9* led to a significantly increased number of invasive and migratory cells in the intervention group [24,34,49]. On the contrary, overexpression of *HOXA5*, *A10* and *D8* resulted in a decreased number of invasive and migratory cells in the intervention group [41,52,55], (Table 2 and Table 3). In vivo findings agreed with the in vitro results regarding *HOXA10* as mice who were injected with cells overexpressing *A10* developed fewer metastases than the control group indicating a protective role of *HOXA10* in CRC progression [55,56]. On the other hand, altered expression of *HOXB8* and *D9* showed the metastasis- promoting effects of these genes in vivo [24,28], (Table 3). The expression of important EMT markers (E-cadherin and vimentin) known to increase the invasive behaviour of cancer cells facilitating metastasis, was altered between the intervention and control group. Specifically, downregulation of E-cadherin with subsequent upregulation of vimentin was observed in the studies investigating metastasis-enhancer *HOX* genes (*A6*, *D9*, *B9*) [24,34,48] whereas the opposite pattern was observed for the metastases-suppressors ones (A5, A10, D8) [41,52,55] (Table 2 and Table 3). Three studies reported findings regarding the role of *HOXB9* in CRC progression; however, the results are contradictory with Zhan et al. showing a metastatic promoting function whereas Huang et al. reported a tumour-suppressive function [46,47,48].

## 4. Discussion

In recent years with the change in people’s lifestyle and dietary factors, the incidence of CRC has been increasing, especially in the younger population, making this disease a public health burden [57]. Since the recurrence and development of distant metastases are the major causes of cancer-related mortality, it is crucial to investigate and discover new molecular markers that contribute to CRC aggressiveness and which may affect survival. We conducted this systematic review to investigate the clinicopathological and prognostic significance of *HOX* genes in CRC and determine the impact of their altered expression in CRC disease progression.

The present systematic review indicates that *HOX* genes become dysregulated in CRC in comparison with normal tissue and are a diverse group of genes, as some may favour disease progression, whereas others act as tumour suppressors in CRC. The combination of clinical and preclinical findings of the studies included revealed that *HOXA3*, *A9*, *B7*, *B8*, *C6*, *C11* and *D9* were found to be upregulated in CRC tissues [24,25,26,36,40,43]. Their high expression was correlated with adverse clinicopathological characteristics of CRC and worse survival outcomes suggesting an oncogenic role which was supported by the in vitro and in vivo experimental observations. On the other hand, *HOXB13* and *HOXD10* were found to be downregulated in CRC, and preclinical studies indicated a protective role towards disease progression [31,44]. Among the dysregulated *HOX* genes, most of them favour an oncogenic behaviour promoting disease progression, rather than acting as tumour suppressors. Similar findings with our study were reported by a recent systematic review by Jin et al. on *HOX* genes in gastric cancer (GC) which demonstrated diversity in the dysregulation profile of *HOX* genes with most of them acting as potential oncogenes and are associated with worse disease characteristics and worse OS [58].

HOXB9 was the most frequently investigated protein and from our post-hoc meta-analysis, we found that HOXB9 high expression was associated with an increased risk for metastatic disease indicating that it may predispose to worse OS. However, studies (n = 5) reported contradictory findings regarding its prognostic role in CRC with Carbone et al. [42], Hoshino et al. [46] and Huang et al. [48] showing a negative association with survival outcomes whereas Song et al. [38] and Zhan et al. [47] reported a positive one. A post-hoc meta-analysis regarding HOXB9 expression and OS could not be conducted due to the insufficient data provided by the studies. Similarly, experimental findings were also opposing between studies regarding its tumour promoting or suppressive role, highlighting that HOX proteins may also have a dual role in CRC progression depending on which mechanism is activated that regulates their function [59,60]. For instance, Wan et al. identified the acetylation of HOXB9 as an important post-translational modification which caused suppression of transcription of the HOXB9 target gene Jumonji domain-containing protein 6 (JMJD6), leading to the inhibition of tumour growth and the migration of lung adenocarcinoma cells, in vitro and in vivo [61]. HOXB9 acetylation was also shown by Song et al. to potentially be responsible for the HOXB9 potential protective role in CRC progression [38,61]. Another potential explanation for the contradictory findings between studies could be the different methodological approaches and categorization of high and low HOXB9 expression groups. For instance, Zhan et al. and Huang et al. evaluated using IHC the association of HOXB9 expression levels with OS. Both studies used IHC as a methodological approach; however, the comparison groups categorized based on IHC staining intensity was different between studies.

HOX proteins contribute to a plethora of functionalities and can be regulated by transcriptional expression, regulating micro-RNAs and post-translational modifications that add complexity in understanding their role [62]. Therefore, the exact mechanism of how HOX proteins promote CRC growth, invasion and metastasis has not yet been elucidated. HOX proteins, as conserved developmental proteins, have the ability to control various cellular functions responsible for cell survival and in many cancers seem to participate in cell proliferation [6]. In CRC Liao et al., showed that HOXB7 could accelerate the transition from G1 to S phase in the cell cycle through the activation of the PI3K/AKT and MAPK pathways resulting in the upregulation of cyclin D1 [26]. Additionally, Zhang et al. found that HOXA3 can serve as an apoptosis-suppressor for cancer development through regulation of apoptosis-related factors (Bcl-2 andcaspase 3) and the activation of the EGFR/Ras/Raf/MEK/ERK pathway [36]. CRC progression to invasive and metastatic disease is characterised by the EMT process, which involves the transition of the stationary cancerous epithelial cells into motile mesenchymal ones enabling them to detach and metastasise [5]. HOX proteins have been found to play an essential role in the EMT, promoting cell invasion and migration. In CRC, Liu et al. reported that HOXD9 promoted CRC cell invasion and migration through enhancing EMT by upregulating vimentin while downregulating E-cadherin [24]. This study also showed that HOXD9 might promote cell invasion and migration through the transforming growth factor-beta (TGF-β) pathway, which is an important pathway in the EMT process in CRC [5]. Our post-hoc meta-analysis identified that HOXB9 was significantly associated with the presence of metastatic disease. There is emerging evidence supporting the role of HOXB9 as a promoter of tumour invasion and metastasis by activating the EMT process through important pathways such as the TGF-β1/Smad2/Slug signalling pathway [63].

Angiogenesis plays a vital role in the progression of cancer, and various HOX proteins have been shown to function in promoting the formation of new vessels in solid tumours by upregulating angiogenic genes [6,63]. In CRC, Hoshino et al. showed that overexpression of HOXB9 resulted in upregulation of angiogenic factors such as interleukin 8 (IL8) and vascular endothelial growth factor (VEGF) both in vitro and in vivo [46]. HOXB9 was also found to be of important clinical significance, as patients with high expression levels appeared to respond better to anti-angiogenic therapy with bevacizumab demonstrating longer OS and DFS in comparison with those who had low HOXB9 levels [46]. Interestingly, Carbone et al. reported the same effect of HOXB9 in the expression of angiogenic factors as Hoshino et al.; however, in vivo models showed that HOXB9 positive nude mice showed resistance to treatment with bevacizumab [42]. Either way, both studies demonstrate that HOXB9 could serve as a potential marker for selecting treatment with anti-angiogenic chemotherapeutic drugs. The possible synergistic role of *HOX* genes modulation with chemotherapy treatment was shown by Yuan et al. for HOXA10, which was found to promote tumour progression in vitro and knockdown resulted in increased sensitivity to 5-Fluorouracil therapy in vitro and in vivo [35].

This systematic review is the first to provide cumulative current evidence regarding the role of *HOX* genes and their protein products in CRC progression, their clinicopathological and prognostic significance. This study outlines the heterogeneity among studies, as many have only investigated a specific *HOX* gene out of the 39 in the human genome. Since the use of *HOX* genes as future biomarkers in CRC has recently started to attract research interest, further studies are warranted on the subject to fully explore the function of each *HOX* gene. This systematic review showed that few studies had been conducted to date that combine clinical and preclinical data (in vitro and in vivo) to thoroughly investigate the clinicopathological and functional role of a *HOX* gene in CRC progression. Moreover, studies demonstrated diversity in the study population characteristics included as well as variability in methodological approaches used. For instance, population characteristics varied between studies in terms of neoadjuvant chemotherapy administration. Furthermore, there was no established 15 standardized cut-off point between a high and low expression level and inconsistent criteria were used between studies to investigate the clinicopathological and prognostic role of the *HOX* gene of interest. It is worth highlighting the lack of power sample size reporting in both clinical and preclinical studies, as well as sample size information in some studies. For reasons which include the diversity between studies and the lack of detailed and robust data a meta-analysis could be conducted only for *HOXB9* on specific outcomes.

## 5. Conclusions

In conclusion, this review provides for the first time systematic evidence that *HOX* genes are dysregulated in patients with CRC and their aberrant expression is related to clinicopathological characteristics and survival. Moreover, this systematic review shows that altered expression of *HOX* genes affects CRC progression in vitro and in vivo. These findings suggest that *HOX* genes may serve as potential biomarkers in CRC and their differential expression may be a candidate hallmark for survival outcomes [64]. The potential clinical application of the findings of this review is that *HOX* genes may be considered as future targets for the development of anticancer therapeutic agents. HOXB9 protein overexpression was identified to be associated with the presence of metastatic disease indicating that it may be a critical transcription factor in CRC. Nevertheless, due to the complexity and heterogeneity of the *HOX* gene family, further well-conducted and even larger-scale or multicentre clinical and preclinical studies with robust methodology are needed to elucidate the role of each gene and especially *HOXB9* in CRC thus determining the validity of their role as potential biomarkers or therapeutic targets in CRC.

## Figures and Tables

**Table 1:** Included studies reporting on clinicopathological and prognostic significance of *HOX* genes in CRC. *: *p* < 0.05, **: *p* < 0.01, ***: *p* < 0.001, ↑: increased, ↓: decreased. Arrows without a * or NR symbol represent findings that are characterized as borderline significant with a *p*-value ranging between 0.051 and 0.1.

Author (Year)	Gene	Patients (%M)	Age (ys)	Stage	FUP (m) (max)	Sample	Methods	DE (C vs. N)	HOX Overexpression Association with Clinicopathological Characteristics (Positive or Negative)								DFS (High vs. Low Expression)	OS (High vs. Low Expression)
									Age	Sex	T	N	M	S	G	CEA		
Liu et al. [24] (2020)	*HOXD9*	100 (59%)	NR	I–IV	NR	FFPE	IHC	↑ ***	NS	NS	NS	↑ *	NR	↑ *	↑ ***	NR	NR	5y: Worse (*p* = 0.000)
Cui et al. [25] (2019)	*HOXC11*	265 (NR)	NR	NR	NR	NR	Data mining	↑ *	NR	NR	NR	NR	NR	NR	NR	NR	NR	10y: Worse (*p* = 0.021)
Ying et al. [28] (2019)	*HOXB8*	80 (59%)	NR	I–IV	120	NR	qRT-PCR	↑ *	NS	NS	NR	↑	NR	↑	NS	NR	NR	10y: Worse (*p* = 0.048)
		510 (NR)	NR	NR	120	NR	Data mining	↑ ***	NS	NS	↑	NS	↑	NS	NS	NR	10y: Worse (*p* = 0.047)	10ys: Worse (*p* = 0.013)
Wu et al. [34] (2018)	*HOXA6*	16 (63%)	49–80	NR	NR	NR	qRT-PCR	↑ *	NR	NR	NR	NR	NR	NR	NR	NR	NR	NR
Yuan et al. [35] (2018)	*HOXA10*	85 (58%)	26–80	II–IV	60	FFPE	IHC	↑ ***	NS	NS	NS	NS	NR	NS	NS	NS	5y: Worse (HR = 4.485, 95%CI:1.163–17.829, *p* = 0.015)	NR
Tatangelo et al. [37] (2018)	*HOXA13*	82 (54%)	50–91	I–IV	NR	FFPE	IHC	↑ (NR)	NS	NS	NS	NS	NR	NR	NS	NS	NR	NR
	*HOXB13*							↑ (NR)	↑	↑	NS	↑ **	NR					
	*HOXC13*							↑ (NR)	NS	NS	NS	↑	NR					
	*HOXD13*							↑ (NR)	NS	NS	NS	NS	NR					
Song et al. [38] (2018)	*AcK27-HOXB9*	90 (51%)	24–90	I–IV	73	FFPE	IHC	↓ ***	↑ *	NS	NS	NS	NR	↓ *	NR	NR	NR	5y: Better (*p* = 0.0007)
Bhatlekar et al. [39] (2018)	*HOXA4*, *HOXD10*	3 (NR)	NR	NR	NR	FT	qRT-PCR/IHC	↑ (NR)	NR	NR	NR	NR	NR	NR	NR	NR	NR	NR
Watanabe et al. [40] (2017)	*HOXA9*	231 (58.9%)	NR	I–IV	100	FT FFPE	qRT-PCR/IHC	↑ ***	NS	NS	NS	↑ *	NS	↑ *	NR	NR	NR	5y: NS (*p* = 0.80)
Mansour et al. [41] (2017)	*HOXD8*	26 (NR)	30–60	II–IV	NR	FT	qRT-PCR and data mining	↓ *	NR	NR	NR	NR	NR	NR	NR	NR	NR	NR
Zhang et al. [36] (2017)	*HOXA3*	232 (61%)	NR	I–IV	140	FFT	qRT-PCR	↑ **	NR	NR	NR	NR	NR	↑ **	NR	NR	10y: Worse (*p* = 0.022)	10y: Worse (*p* = 0.024)
Carbone et al. [42] (2017)	*HOXB9*	58 (53%)	25–84	I–IV	NR	FFPE	IHC	NR	NS	NR	↑ *	NR	↑	NR	NR	NR	5y: Worse, (HR = 2.552, 95%CI:1.180–5.518, *p* = 0.017)	NR
Ji et al. [43] (2016)	*HOXC6*	462 (61%)	NR	I–IV	84	FFPE	IHC	↑ ***	NS	NS	↑ ***	↑ ***	NS	NR	NS	NS	NS	5y: Worse, (HR = 2.14, 95%CI: 1.487–3.088, *p* < 0.001)
Wang et al. [44] (2016)	*HOXD10*	126 (59%)	NR	I–III	NR	FFT	qRT-PCR/IHC	↓ **	NR	NR	NR	↓ **	NR	NR	NR	NR	NR	NR
Shen et al. [45] (2016)	*HOXB8*	30 (63%)	20–90	I–IV	NR	FFT	qRT-PCR/WB	EQ	NS	NS	NS	NS	NS	NS	NS	NR	NR	NR
Hoshino et al. [28] (2014)	*HOXB9*	93 (NR)	NR	II–III	NR	FFT FFPE	qRT-PCR/IHC	↑ (NR)	NR	NR	NR	NR	NR	NR	↑ ***	NR	NS	5y: Worse (*p* = 0.038)
Zhan et al. [47] (2014)	*HOXB9*	63 (54%)	24–90	I–IV	73	FFPE	IHC	NR	NR	NS	NS	NS	NS	NS	↓ *	NR	NR	5y: Better (*p* = 0.040)
Huang et al. [48] (2014)	*HOXB9*	128 (47%)	NR	I–IV	60	FFT FFPE	IHC/WB	↑ *	NS	NS	NS	↑ *	↑ **	↑	NS	NS	NR	5y: Worse (*p* = 0.013)
Liao et al. [26] (2011)	*HOXB7*	224 (57%)	23–86	I–IV	87	FFT FFPE	qRT-PCR/IHC	↑ (NR)	NS	NS	↑ *	↑	↑ *	↑ ***	NR	NR	NR	5y: Worse, (HR = 2.279, 95%CI: 1.062–2.687, *p* = 0.027)
Kanai et al. [27] (2010)	*All HOX*	40 (68%)	48–89	I–IV	NR	FFT	qRT-PCR	↑*: A9,B3, B8,B9 ↓**: B2,B13, D1,D3, D4,D8, D12	NR	NR	NR	↑* A3D1	NR	NR	NR	NR	NR	NR
Cantile et al. [29] (2009)	*HOXD13*	48 (NR)	NR	NR	NR	FFT	qRT-PCR	↑ ***	NR	NR	NR	NR	NR	NR	NR	NR	NR	NR
Groene et al. [30] (2006)	*HOXA9*	36 (50%)	NR	II–III	NR	FFT	qRT-PCR	NR	NR	NR	NR	NR	NR	↑ *	NR	NR	NR	NR
Jung et al. [31] (2005)	*HOXB13*	53 (NR)	NR	NR	NR	FFT	qRT-PCR	↓ (NR)	NR	NR	NR	NR	NR	NR	NR	NR	NR	NR
Toiyama et al. [32] (2005)	*HOXA4*	4	40–68	NR	NR	FT	qRT-PCR	↓ **	NR	NR	NR	NR	NR	NR	NR	NR	NR	NR
Vider et al. [33] (1997)	*HOXB5*, *B6*, *B7*, *B8*, *B9*, *C9*	11 (NR)	NR	NR	NR	FFT	qRT-PCR	↑ (NR)	NR	NR	NR	NR	NR	NR	NR	NR	NR	NR

**%M**: percentage of male patients, **FUP**: Follow up, **DE**: Differential Expression, **C**: Cancerous tissue, **N**: Normal colon tissue, **T**: Tumour depth, **N**: Lymph node status, **M**: presence of metastatic disease, **S**: Stage, **G**: Grade, **CEA**: Carcinoembryonic antigen, **DFS**: Disease-Free Survival, **OS**: Overall Survival, **NR**: Not Reported, **EQ**: Equivocal findings (defined as the difference in DE pattern between mRNA and protein expression), **FFPE**: Fixed Formalin Paraffin-Embedded, **FT**: Fresh Tissue, **FFT**: Fresh Frozen Tissue, **IHC**: Immunohistochemistry, **WB**: Western Blot, **NS**: Not Significant, **RT-qPCR**: Real-Time Quantitative Polymerase Chain Reaction, **HR**: Hazard Ratio, **CI**: Confidence Intervals, *: *p* < 0.05, **: *p* < 0.01, ***: *p* < 0.001.

**Table 2:** Summary of findings of the included studies that performed only in vitro experiments on the functional role of *HOX* genes dysregulation in CRC progression. *: *p* < 0.05, **: *p* < 0.01, ***: *p* < 0.001, ↑: increased, ↓: decreased.

Author (Year)	Gene	Intervention	Outcomes (Intervention vs. Control Cell Line Group)				
			PR	CLF	AP	INV	MIGR
Studies Performed in vitro Experiments, only							
Cui et al. [25] (2019)	*HOXC11*	KD	↓ *	NR	↑ *	NR	NR
Li et al. [49] (2019)	*HOXB2*	OE	↑ ***	NR	NR	↑ **	↑ **
Wu et al. [34] (2018)	*HOXA6*	OE	↑ ***	↑ **	↓ **	↑ ***	↑ ***
Li et al. [50] (2018)	*HOXA1*	KD	↓ **	NR	NR	↓ **	NR
Watanabe et al. [40] (2018)	*HOXA9*	KD	NS	NR	NR	NR	NR
Bhatlekar et al. [51] (2018)	*HOXA4* *HOXA9*	KD	↓ **	↓ **	NR	NR	NR
Mansour et al. [41] (2017)	*HOXD8*	OE	↓ *	↓ *	↑ *	↓ *	NR
Han et al. [52] (2017)	*HOXA5*	OE	↓ **	↓ **	NR	↓ **	↓ **
Chen et al. [53] (2016)	*HOXD3*	KD	↓ **	↓ **	↑ **	NR	NR
Kasiri et al. [54] (2013)	*HOXC13*	KD	↓ *	NR	↑ (NR)	NR	NR
Jung et al. [31] (2005)	*HOXB13*	OE	↓ (NR)	NR	NR	NR	NR

**PR**: proliferation, **CLF**: colony formation, **AP**: apoptosis, **INV**: invasion, **MIGR**: migration, **OE**: overexpression, **KD**: knockdown, **NR**: not reported.

**Table 3:** Summary of findings of the included studies that performed both in vitro and in vivo experiments on the functional role of *HOX* genes dysregulation in CRC progression. *: *p* < 0.05, **: *p* < 0.01, ***: *p* < 0.001, ↑: increased, ↓: decreased.

Author (Year)	Gene	Intervention	Outcomes (Intervention vs. Control Cell Line Group)					Nude Mice (Type, n)	Outcomes (Intervention vs. Control Mice Group)
			PR	CLF	AP	INV	MIGR		
Studies Performed in vitro and in vivo Experiments									
Liu et al. [24] (2020)	*HOXD9*	OE	↑ ***	↑ **	NR	↑ ***	↑ ***	BALB/c (n = NR)	Lung mets: ↑ *** Liver mets: ↑ ***
Ying et al. [28] (2019)	*HOXB8*	KD	↓ **	↓ **	NR	↓ *	↓ **	BALB/c (n = 24)	TV (mm^3^): ↓ ** TW (gr): ↓ ** Liver mets: NS
Zhang et al. [36] (2018)	*HOXA3*	KD	↓ **	↓ **	↑ ***	NR	NR	Nod N = 10	TW (gr): ↓ ***
Yuan et al. [35] (2018)	*HOXA10*	KD	NR	↓ (NR)	↑ (NR)	NR	NR	BALB/c (n = 10)	TV (mm^3^): ↓ **
Ji et al. [43] (2016)	*HOXC6*	KD	↓ ***	↓ ***	NS	NR	NR	Nu/Nu (n = 8)	TS (cm): ↓ *
Sun et al. [55] (2016)	*HOXA10*	OE	NR	NR	NR	↓ *	NR	BALB/c (n = 10)	Lung mets: ↓ **
Hoshino et al. [46] (2014)	*HOXB9*	OE	NR	NR	NR	NR	NR	BALB/c (n = 8)	TV (mm^3^): ↑ *** TW (gr): ↑ ***
Zhan et al. [47] (2014)	*HOXB9*	OE	↓ **	NR	NR	↓ **	↓ **	BALB/c (n = 19)	TW (gr): ↓ ** Lung mets: ↓ (NR) (37.5% vs. 50%) Liver mets: ↓ (NR) (37.5% vs. 70%)
Huang et al. [48] (2013)	*HOXB9*	KD	NR	NR	NR	↓ *	↓ *	BALB/c (n = 24)	Lung mets: ↓ (NR) (0% vs. 56%) Liver mets: ↓ (NR) (12% vs. 81%)
Liao et al. [26] (2011)	*HOXB7*	OE	↑ *	↑ **	NR	NR	NR	BALB/c (n = 10)	TV (mm^3^): ↑ *
Ghoshal et al. [56] (2010)	*HOXB13*	OE	↓ **	↓ **	NR	NR	NR	NR	TW (gr): ↓ *** TV (mm^3^): ↓ ***

**PR**: proliferation, **CLF**: colony formation, **AP**: apoptosis, **INV**: invasion, **MIGR**: migration, **OE**: overexpression, **KD**: knockdown, **NR**: not reported, **NS**: non-significant, **TV**: tumour volume, **TW**: tumour weight, **TS**: tumour size, *: *p* < 0.05, **: *p* < 0.01, ***: *p* < 0.001, ↑: increased, ↓: decreased.

## Data Availability

All data presented in this study are included in the paper and its supplementary documents.

## Supplementary Materials

The following are available online at https://www.mdpi.com/article/10.3390/ijms222413429/s1.
