# Supplementary material for: A Systematic Review on HOX Genes as Potential Biomarkers in Colorectal Cancer: An Emerging Role of HOXB9
Source: Int J Mol Sci. 2021 Dec 14;22(24):13429. doi: 10.3390/ijms222413429 (PMC8707253; doi:10.3390/ijms222413429)
Supplement: Supplementary file 1 [file ijms-22-13429-s001.zip › ESM_1.pdf]

SEARCH STRATEGY ALGORITHMS

Strategy for Pubmed (field labels were used to restrict specific terms/phrases to Medical Subject Headings [MeSH] or by publication type [pt] and title/abstract [tiab] fields) No limitations were used.

1. "genes, homeobox"[MeSH Terms] OR "Hox"[tiab] OR "homeobox"[tiab] OR "Homeoboxes"[tiab] OR "Homeo Box"[tiab] OR "Homeo Boxes"[tiab] OR "Homeotic"[tiab]
2. "colon"[MeSH Terms] OR "colon"[tiab] OR "colorectal"[tiab] OR "rectal"[tiab] OR "rectum"[MeSH Terms] OR "rectum"[tiab] OR "gastrointestinal"[tiab] OR "colonic"[tiab] OR "intestinal"[tiab] OR "intestine"[tiab] OR "intestines"[MeSH Terms] OR "intestines"[tiab] OR "bowel"[tiab] OR "digestive system"[MeSH Terms] OR "digestive"[tiab] OR "cecum"[MeSH Terms] OR "cecum"[tiab] OR "caecum"[tiab] OR "cecal"[tiab] OR "caecal"[tiab] OR "anal canal"[MeSH Terms] OR "anal"[tiab] OR "anus"[tiab] OR "colon, sigmoid"[MeSH Terms] OR "sigmoid"[tiab] OR "rectosigmoid"[tiab]
3. "neoplasms"[MeSH] OR "neoplasms"[tiab] OR "neoplasm"[tiab] OR "cancer"[tiab] OR "cancers"[tiab] OR "tumour"[tiab] OR "tumours"[tiab] OR "tumor"[tiab] OR "tumors"[tiab] OR "neoplasia"[tiab] OR "malignancy"[tiab] OR "malignancies"[tiab] OR "malignant"[tiab] OR "carcinoma"[MeSH Terms] OR "carcinoma"[tiab] OR "carcinomas"[tiab] OR "neoplastic"[tiab]
4. "editorial"[pt] OR "letter"[pt] OR "correspondence as topic"[MeSH Terms] OR "review"[pt] OR "review literature as topic"[MeSH Terms]

Final search for research questions: #1 AND #2 AND #3 NOT #4

Strategy for Embase (field labels were used to restrict specific terms/phrases by publication type and title/abstract/keyword [ti,ab,kw] fields). No limitations were used.

1. "Hox":ti,ab,kw OR "homeobox":ti,ab,kw OR "Homeoboxes":ti,ab,kw OR "Homeo Box":ti,ab,kw OR "Homeo Boxes":ti,ab,kw OR "Homeotic":ti,ab,kw
2. "colon":ti,ab,kw OR "colorectal":ti,ab,kw OR "rectal":ti,ab,kw OR "rectum":ti,ab,kw OR "gastrointestinal":ti,ab,kw OR "colonic":ti,ab,kw OR "intestinal":ti,ab,kw OR "intestine":ti,ab,kw OR "intestines":ti,ab,kw OR "intestines":ti,ab,kw OR "bowel":ti,ab,kw OR "digestive system":ti,ab,kw OR "digestive":ti,ab,kw OR "cecum":ti,ab,kw OR "caecum":ti,ab,kw OR "cecal":ti,ab,kw OR "caecal":ti,ab,kw OR "anal":ti,ab,kw OR "anus":ti,ab,kw OR "sigmoid":ti,ab,kw OR "rectosigmoid":ti,ab,kw
3. "neoplasms":ti,ab,kw OR "neoplasm":ti,ab,kw OR "cancer":ti,ab,kw OR "cancers":ti,ab,kw OR "tumour":ti,ab,kw OR "tumours":ti,ab,kw OR "tumor":ti,ab,kw OR "tumors":ti,ab,kw OR "neoplasia":ti,ab,kw OR "malignancy":ti,ab,kw OR "malignancies":ti,ab,kw OR "malignant":ti,ab,kw OR "carcinoma":ti,ab,kw OR "carcinomas":ti,ab,kw OR "neoplastic":ti,ab,kw
4. "editorial":ti,ab,kw OR "letter":ti,ab,kw OR "correspondence":ti,ab,kw OR "review":ti,ab,kw

Final search for research questions: #1 AND #2 AND #3 NOT #4

Strategy for Web of Sciences (All terms were searched in “Topic”). No limitations were used.

1. "Hox" OR "homeobox" OR "Homeoboxes" OR "Homeo Box" OR "Homeo Boxes" OR "Homeotic"
2. "colon" OR "colorectal" OR "rectal" OR "rectum" OR "gastrointestinal" OR "colonic" OR "intestinal" OR "intestine" OR "intestines" OR "intestines" OR "bowel" OR "digestive system" OR "digestive" OR "cecum" OR "caecum" OR "cecal" OR "caecal" OR "anal" OR "anus" OR "sigmoid" OR "rectosigmoid"
3. "neoplasms" OR "neoplasm" OR "cancer" OR "cancers" OR "tumour" OR "tumours" OR "tumor" OR "tumors" OR "neoplasia" OR "malignancy" OR "malignancies" OR "malignant" OR "carcinoma" OR "carcinomas" OR "neoplastic"
4. "editorial":pt OR "letter":pt OR "correspondence":pt OR "review":pt

Final search for research questions: #1 AND #2 AND #3 NOT #4

Strategy for Cochrane Library

Default search fields (Title, Abstract, Keywords) was used for #1, #2, #3.

Publication type fields was used for #4

No limitations were used.

1. MeSH descriptor: [Genes, Homeobox] explode all trees
2. MeSH descriptor: [Colonic Neoplasms] explode all trees
3. MeSH descriptor: [Rectal Neoplasms] explode all trees
4. MeSH descriptor: [Sigmoid Neoplasms] explode all trees
5. MeSH descriptor: [Colorectal Neoplasms] explode all trees
6. MeSH descriptor: [Intestinal Neoplasms] explode all trees

7. "Hox" OR "homeobox" OR "Homeoboxes" OR "Homeo Box" OR "Homeo Boxes" OR "Homeotic"
8. "colon" OR "colorectal" OR "rectal" OR "rectum" OR "gastrointestinal" OR "colonic" OR "intestinal" OR "intestine" OR "intestines" OR "intestines" OR "bowel" OR "digestive system" OR "digestive" OR "cecum" OR "caecum" OR "cecal" OR "caecal" OR "anal" OR "anus" OR "sigmoid" OR "rectosigmoid"
9. "neoplasms" OR "neoplasm" OR "cancer" OR "cancers" OR "tumour" OR "tumours" OR "tumor" OR "tumors" OR "neoplasia" OR "malignancy" OR "malignancies" OR "malignant" OR "carcinoma" OR "carcinomas" OR "neoplastic"

10. #1 OR #7
11. #2 OR #3 OR #4 OR #5 OR #6
12. #8 AND #9
13. #11 OR #12
14. Final search for research questions: #10 AND #13
