# Supplementary material for: A Systematic Review on HOX Genes as Potential Biomarkers in Colorectal Cancer: An Emerging Role of HOXB9
Source: Int J Mol Sci. 2021 Dec 14;22(24):13429. doi: 10.3390/ijms222413429 (PMC8707253; doi:10.3390/ijms222413429)
Supplement: Supplementary file 1 [file ijms-22-13429-s001.zip › Table S3.pdf]

Author (year)	Gene	Cell lines	Intervention	Endpoint assays
**Cui *et al*** [25] **(2019)**	*HOXC11*	SW480	siRNA (KD)	Proliferation (CCK-8)<br>Flow cytometry
**Li *et al*** [49] **(2019)**	*HOXB2*	SW620<br>SW480	Plasmid (OE)	Proliferation (MTT)<br>Wound healing<br>Transwell invasion
**Wu *et al*** [34] **(2018)**	*HOXA6*	HT29<br>Caco-2	Plasmid (OE)<br>ShRNA (KD)	Proliferation (CCK-8)<br>Colony formation<br>Transwell invasion/migration<br>Flow cytometry
**Li *et al*** [50] **(2018)**	*HOXA1*	SW620	siRNA (KD)	Proliferation (CCK-8)<br>Transwell invasion
**Watanabe *et al*** [40] **(2018)**	*HOXA9*	HCT116, LoVo<br>RKO, LS174T<br>Colo205<br>Colo201 SW620,<br>LS180 SW83,<br>SW480 HCT15,<br>SW480	SiRNA (KD)	Proliferation (trypan blue)
**Bhatlekar *et al*** [51] **(2018)**	*HOXA4*<br>*HOXA9*	SW480<br>HT29	siRNA (KD)	Proliferation (WST-1)<br>Colony formation
**Mansour *et al*** [41] **(2017)**	*HOXD8*	HCT116, DLD-1<br>HT29, SW620<br>SW1080	Retrovirus (OE)	Proliferation (MTT)<br>Colony formation<br>Apoptosis<br>Transwell invasion
**Han *et al*** [52] **(2017)**	*HOXA5*	HCT116<br>HT29	Plasmid (OE)	Proliferation (CCK-8)<br>Colony formation<br>Transwell invasion/migration
**Chen *et al*** [53] **(2016)**	*HOXD3*	RKO	Lentivirus (KD)	Proliferation (MTT)<br>Colony formation<br>Flow cytometry
**Kasiri *et al*** [54] **(2013)**	*HOXC13*	SW480	Antisense nucleotide (KD)	Proliferation (MTT)<br>Flow cytometry
**Jung *et al*** [31] **(2005)**	*HOXB13*	HCT116, LoVo,<br>SW480, Caco-2	Plasmid (OE)	Proliferation (MTT)
**KD**: Knockdown, **OE**: Overexpression, **NR**: Not Reported, **CCK-8**: Cell Counting Kit 8, **MTT**: 3-(4,5-dimethylthiazol-2-yl)-2,5-diphenyltetrazolium bromide				

Table S3. Summary of characteristics of the included studies that performed only *in vitro* experiments on the functional role of *HOX* genes dysregulation in CRC progression.
