# Supplementary material for: A Systematic Review on HOX Genes as Potential Biomarkers in Colorectal Cancer: An Emerging Role of HOXB9
Source: Int J Mol Sci. 2021 Dec 14;22(24):13429. doi: 10.3390/ijms222413429 (PMC8707253; doi:10.3390/ijms222413429)
Supplement: Supplementary file 1 [file ijms-22-13429-s001.zip › Table S4.pdf]

Author (year)	Gene	Cell lines	*In vitro* Intervention	*In vitro* Endpoint assays	*In vivo* Intervention
**Liu *et al*** [24] **(2020)**	*HOXD9*	LoVo, SW116	Lentivirus (KD) Plasmid (OE)	Proliferation (CCK-8) Wound healing Transwell invasion	Caecal injection
**Ying *et al*** [28] **(2019)**	*HOXB8*	HCT116	Lentivirus (KD)	Proliferation (CCK-8) Colony formation Transwell invasion/migration	Flank SC & spleen injection
**Zhang *et al*** [36] **(2018)**	*HOXA3*	HCT116 HT29	siRNA (KD)	Proliferation (MTT) Colony formation Flow cytometry	Flank SC injection
**Yuan *et al*** [35] **(2018)**	*HOXA10*	LoVo HT29	Lentivirus (KD)	Proliferation (MTT) Colony formation Flow cytometry	Flank SC injection
**Ji *et al*** [43] **(2016)**	*HOXC6*	HCT116	Lentivirus (KD)	Proliferation (MTT) Colony formation Flow cytometry	Forelimb SC injection
**Sun *et al*** [55] **(2016)**	*HOXA10*	SW480	Lentivirus (OE) siRNA (KD)	Transwell invasion	Flank SC injection
**Hoshino *et al*** [46] **(2014)**	*HOXB9*	HCT116	Plasmid (OE) Lentivirus (KD)	Proliferation (MTT)	Flank SC injection
**Zhan *et al*** [47] **(2014)**	*HOXB9*	HCT116	Plasmid (OE) siRNA (KD)	Proliferation (WST-1) Transwell migration/invasion	Flank SC injection
**Huang *et al*** [48] **(2013)**	*HOXB9*	LoVo SW620	Retrovirus (KD) Plasmid (OE)	Wound healing Transwell invasion/migration	Orthotopic implantation
**Liao *et al*** [26] **(2011)**	*HOXB7*	HCT116, Ls174t, SW480, SW620, DLD-1	Plasmid (OE) Retrovirus (KD)	Proliferation (MTT) Colony formation	Flank SC injection
**Ghoshal *et al*** [56] **(2010)**	*HOXB13*	HCT116 RKO	Plasmid (OE)	Proliferation (MTT) Colony formation	Flank SC injection

KD: Knockdown, **OE**: Overexpression, **NR**: Not Reported, **CCK-8**: Cell Counting Kit 8, **MTT**: 3-(4,5-dimethylthiazol-2-yl)-2,5-diphenyltetrazolium bromide, **SC**: subcutaneous

Table S4. Summary of characteristics of the included studies that performed both *in vitro* and *in vivo* experiments on the functional role of *HOX* genes dysregulation in CRC progression.
